# Lipid yield from the diatom *Porosira glacialis* is determined by solvent choice and number of extractions, independent of cell disruption

**DOI:** 10.1038/s41598-020-79269-z

**Published:** 2020-12-17

**Authors:** Jon Brage Svenning, Lars Dalheim, Terje Vasskog, Lucie Matricon, Birthe Vang, Ragnar Ludvig Olsen

**Affiliations:** 1 Norwegian College of Fishery Science, UiT The Arctic University of Norway, 9037 Tromsø, Norway; 2 Department of Pharmacy, Faculty of Health Sciences, UiT The Arctic University of Norway, 9037 Tromsø, Norway; 3 Nofima AS, Muninbakken 9-13, Breivika, 9019 Tromsø, Norway

**Keywords:** Industrial microbiology, Microbiology techniques

## Abstract

Cell wall disruption is necessary to maximize lipid extraction yields in conventional species of mass-cultivated microalgae. This study investigated the effect of sonication, solvent choice and number of extractions on the lipid yield, lipid class composition and fatty acid composition of the diatom *Porosira glacialis.* For comparison, the diatom *Odontella aurita* and green alga *Chlorella vulgaris* were included in the study*.* Sonication effectively disrupted *P. glacialis* cells, but did not increase the total lipid yield compared to physical stirring (mixing). In all three microalgae, the content of membrane-associated glyco- and phosopholipids in the extracted lipids was strongly dependent on the solvent polarity. A second extraction resulted in higher yields from the microalgae only when polar solvents were used. In conclusion, choice of solvent and number of extractions were the main factors that determined lipid yield and lipid class composition in *P. glacialis.*

## Introduction

Marine photoautotrophic microalgae are a largely unexploited source of lipids with a wide range of possible uses, such as biodiesel production^1^, fish oil substitution in aquaculture feed^2^ or nutraceuticals for human consumption^3^. The high costs associated with cultivation and extraction of lipids have, however, prevented the production of low-cost products, and the current production of microalgae is limited to high-priced lipophilic products such as pigments, omega-3 supplements or other valuable biomolecules^4–6^. One of the main challenges to reduce the processing cost is to maximize the product recovery from microalgal biomass. In this respect, cell disruption prior to extraction is a prerequisite in frequently mass-cultivated microalgae due to tough cell walls that prevent lipid release^7,8^. Diatoms have silica-based cell walls, which are fragile compared to the tough cell walls associated with microalgae such as *Nannochloropsis* sp.^9^ or *Chlorella* sp.^10^. As a consequence, an efficient extraction from diatom biomass may be less challenging. In addition, cell disruption has been shown to enzymatically release fatty acids from membrane lipids in diatoms. Polyunsaturated fatty acids may then be transformed and degraded by lipoxygenases and lyases to potentially harmful secondary oxidation products, collectively known as oxylipins^11–13^. Removing cell disruption from the oil extraction procedure may therefore avoid reduction in the PUFA content and the need for extensive refinement to remove free fatty acids and oxidation products from the oil.

Of the two most commonly used methods in laboratory settings, Folch’s method^14^ is preferentially used for lipid extraction of marine biomass, as the method of Bligh and Dyer^15^ tends to underestimate the lipid content in lipid-rich marine organisms^16^. In its original design, the Folch method utilizes a mixture of chloroform and methanol as the organic phase. The less toxic dichloromethane functions equally well^17,18^, and is now the preferred choice in most Folch extractions. However, as both methanol and dichloromethane pose potentially serious health hazards, alternative solvents of lower toxicity such as hexane/isopropanol have been suggested^19^. In large-scale industrial production of algal biomass, the traditional methods for lipid extraction become impractical due to the cost and health risks associated with organic solvents. As a result, studies have investigated the use of hexane alone to extract microalgal oils for biodiesel production^20,21^. Compared to the chlorinated solvents, hexane has a lower cost, higher specificity toward lipids of low polarity such as triglycerides, and is less problematic to dispose of. As the diatom studied in this experiment has a high content of polyunsaturated fatty acids bound to an abundance of complex membrane lipids^22,23^, hexane alone is unlikely to provide an efficient extraction. Cell disruption prior to extraction may, however, increase the yield of the more polar lipids when extracting with hexane.

Although most laboratory procedures rely on a single extraction to isolate lipid from a given biomass, one study found that repeating the extraction twice independently of solvent choice significantly increased the lipid yield from the green microalgae *Chlorella* sp.^24^. However, while studies on other microalgae are helpful when developing new methods, the final choice of extraction method is probably species-dependent, due to the highly diverse membrane physiology, morphology and biochemistry within the phytoplankton^25^. *Porosira glacialis,* the diatom used in this study, is a large (Ø > 30 μm), cold-water strain with the potential to convert CO_2_ from flue gas in large-scale photobioreactors into valuable products such as omega-3 fatty acids^26^. The aim of the present study was to investigate how different cell disruption methods affected the integrity of this diatom cultivated in a pilot scale. The lipid yield when using industrially relevant solvents after applying the most destructive disruption technique of the biomass was compared with a relatively gentle mixing more suitable for large scale processing. The lipid classes and the fatty acid composition of the extracted lipids were also determined. For comparison, the green algae *Chlorella vulgaris* and the diatom *Odontella aurita,* both commercially available, were included in the study*.*

## Materials and methods

### Materials

Lyophilized material of *C. vulgaris* (Midsona, Oslo, Norway) was purchased from a local health store. Lyophilized *O. aurita* was obtained from KissPlanet (Gembloux, Belgium). Kristalon Flower was purchased from Yara Norge as, Oslo, Norway. Sodium metasilicate pentahydrate was acquired from Skovly Engros as, Oslo, Norway. Kits for quantifying NO_3_, NO_2_, silicic acid, PO_4_ and NO_4_ were purchased from VWR, Radnor, Pennsylvania, USA. Dichloromethane (99.9%), methanol (99.8%), sulfuric acid (95–97%), hexane (99%), sodium metasilicate pentahydrate (≥ 95%), sodium chloride, isopropanol and lipid standards of diacylglyceryl-trimethylhomoserine (1,2-dipalmitoyl-sn-glycero-3-O-4′-(N,N,N-trimethyl)-homoserine; DGTS), sulfoquinovosyldiacylglycerol (SQDG) and phosphatidylinositol (PI) were purchased from Sigma Aldrich, St. Louis, Missouri, USA. Lipid standards of phosphatidylcholine (1,2-Dimyristoyl-sn-Glycero-3-Phosphatidylcholine; PC), phosphatidylglycerol (1,2-Dimyristoyl-sn-Glycero-3-Phosphatidylglycerol Na Salt; PG), phosphatidylserine (1,2-Dipalmitoyl-sn-Glycero-3-Phosphatidylserine Na salt; PS), phosphatidylethanolamine (1,2-Dimyristoyl-sn-Glycero-3-Phosphatidylethanolamine; PE), hydrogenated monogalactosyl diglyceride (MGDG), hydrogenated digalactosyl diglyceride (DGDG), ergosterol, triolein (TAG), diolein (DAG) and monolein (MAG) were purchased from Larodan AB, Solna, Sweden.

### Diatom strain cultivation and harvesting

The monoculture of *P. glacialis* used in this study was isolated from a sediment sample collected in the Barents Sea in 2014 and identified using light microscopy and SEM imaging^27^. The cultivation was performed in a 300,000-L vertical column photobioreactor placed outdoors, mixed by continuous aeration using pressurized air. The culture was illuminated with LEDs (VIS, PAR radiation) at a mean illumination of ca. 18 μmol m^−2^ s^−1^ with reference to a spherical PAR sensor (Biospherical, QSL-100). The seawater used in the cultivation was collected at 25-m depth, pre-filtered at 1 μm and disinfected using ultraviolet radiation. The cultivation temperature was 6 °C. Inorganic nutrients were added in the form of 0.1 g/l Kristalon flower (14% N, 3.9% P) and sodium metasilicate pentahydrate stock solution (0.1 g/l in H_2_O). The concentration of inorganic nutrients was measured daily using the kits listed above. In order to maintain a nutrient replete environment, the concentrations of N and Si were maintained within 50–150 μM and 20–150 μM, respectively. The culture medium was also enriched with CO_2_ by aerating the culture with flue gas (6–12% CO_2_) to pH < 8.0 on a daily basis. The culture, as part of a longer period of sampling for various experimental work, was maintained in exponential growth at approximately 20 million cells/l by daily cell counts and dilutions. Harvesting was performed by passing the culture through a continuous solid bowl centrifuge (Model PTDC, Nanjing Kingreat Machinery Company, Jiangsu, China) at 835 G and collecting the resulting biomass with a spatula and placing the biomass at − 80 °C while awaiting analysis.

### Evaluation of methods for cell disruption

Thawed biomass of *P. glacialis* was mixed in water (1 mg/ml) and exposed to the following cell disruption methods: Microwave (EV-880MD, Evalet) at 2450 MHz for 3 min and 45 s; sonication at 20 kHz for 3, 5, 10 and 15 min on ice (VC50, Sonics and Materials Inc.), ultrathurax (Polytron PT 1200 E) for 10 min at 25,000 RPM and manual grinding using a PTFE pestle. The effect of lyophilization was also evaluated by freeze-drying *P. glacialis* and re-dissolving 1 mg/ml wet-weight equivalent in water. Following treatment, each method was evaluated by the visual appearance of the biomass in a microscope (Zeiss Axio Vert.A1) at 100 × magnification, and the most efficient method chosen as the cell disruption method prior to lipid extraction.

### Determination of ash-free dry weight

Following freeze-drying, five replicates of 300 mg dry weight (DW) were placed at 105 °C in pre-burned, open aluminum containers for 24 h to determine the DW, and then combusted in a muffle furnace to determine the ash-free dry weight (AFDW).

### Physical treatment and lipid extraction:

The control lipid extraction method used in this experiment was based on the method developed by Folch et al.^14^. Lyophilized biomass was divided into five replicates of 150 mg in 15 ml centrifuge tubes and added 20 volumes (3 ml) of either dichloromethane/methanol (2:1 v/v, DCM/MeOH), hexane/isopropanol (2:1 v/v, hexane/IPA) or hexane. Following the addition of solvent, the samples were subjected to the following treatments: No treatment (control), stirring using a shaker (Heidolph Multireax) at 1000 RPM for 60 min at room temperature (mixing), and sonication at 20 kHz for 10 min (sonication) on ice. The samples were then added 3 ml MiliQ water added 5% NaCl and centrifuged for 5 min at 3000 G, before the organic phase was transferred to a 4 ml vial and evaporated under nitrogen. The extraction procedure was repeated once without physical treatment for each sample, and the yield was determined gravimetrically for each extraction respectively as percent of AFDW. Finally, the samples were dissolved (10 mg/ml) in DCM/MeOH (2:1 v/v) and stored at -80 °C.

### Fatty acid methylation and GC analysis

Fatty acids were methylated using a method developed by Stoffel et al.^28^ with modifications, for a detailed description of the derivatization procedure see Svenning et al.^23^. The fatty acid methyl esters (FAMEs) were analyzed on a GC-FID (Agilent Technologies) coupled to a Select FAME column (length 50 m, ID 0.25 mm and FT 0.25 μm, Agilent J&W Columns). The GC conditions were as follows: Helium was used as the carrier gas at a rate of 1.6 ml/min. The inlet temperature was set to 240 °C (split 1:50), and the FID was set to 250 °C. The oven temperature was programmed to 60 °C for one minute, then increased to 130 °C at a rate of 30 °C/min, then to 195 °C at a rate of 1.3 °C/min, before finally increasing to 240 °C at a rate of 30 °C/min for 10 min. The fatty acids were identified using fatty acids standards quantified by dividing the peak area of the chromatograms with the area of the internal standard (heptadecaenoic acid), and converted to absolute amounts using the slopes calculated from standard curves (triplicates of 7.8125–2000 μg/ml of GLC 502 Free Acids, Nu-Check-Prep, Elysian, MN, USA).

### Lipid class analysis by HPLC

The composition of lipid classes was analyzed using a Waters e2795 separations module, coupled to a Supelcosil™ LC-SI 5 μm (25 cm × 4.6 mm) column (Supelco HPLC products, Bellefonte, PA, USA) set to a working temperature of 40 °C. The HPLC method used was developed by Abreu et al.^29^. Lipids were quantified using a Waters 2424 ELS detector set to gain 100, nebulizer heating level set to 30%, drift tube temperature set to 45 °C and pressure set to 40 PSI. The total run time was 41 min, using the gradient profile and mobile phases listed in Supplementary Table S1. Lipids were quantified based on the peak area in the chromatograms and converted to absolute amounts based on standard curves (triplicates of 12.5–400 μg/ml of the lipid classes listed in “Materials”). All samples and standards were dissolved in mobile phase A/Chloroform (4:1 v/v) prior to analysis.

### Data presentation and statistics

All analyses were performed using 5 replicates and presented as means ± standard deviations, either in tables or as figures with error bars representing one standard deviation. All statistical analyses were prepared using R v3.6.1 (‘Action of the Toes’), making use of the ‘ggplot2’ package, and a range of packages in the Tidyverse. Means of total lipid content, fatty acid and lipid class composition were compared with the pair-wise Tukey test, assuming a normal distribution. Means were determined different at a significance level of < 0.05. All numerical values and methods for hypothesis testing and descriptive statistical procedures are included in the R markdown supplied with this study, see Data availability.

## Results and discussion

### Evaluation of methods for cell disruption

**Figure 1:**
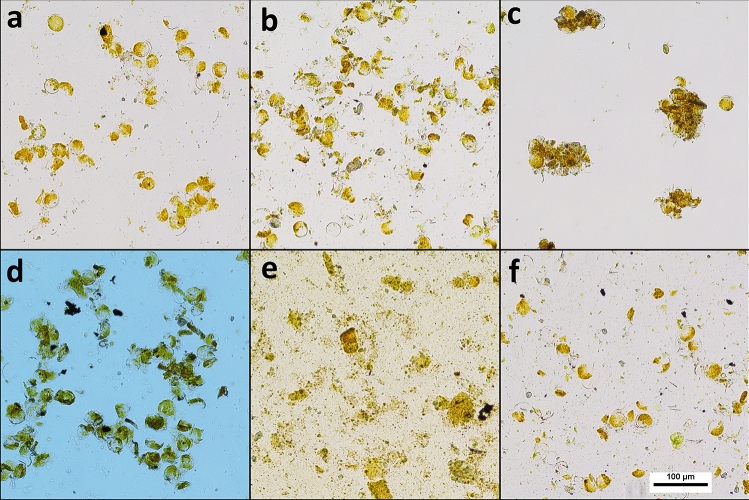
The effect of cell disruption on thawed biomass of *Porosira glacialis*. (**a**) Control, (**b**) manual grinding using a PTFE pestle, (**c**) microwave, (**d**) lyophilization, (**e**) sonication, (**f**) Ultrathurax. All images were captured at 100 × magnification.

Of the five treatments applied for cell disruption, lyophilization (Fig. 1d) was the least effective method compared to the control (Fig. 1a). Sonication (Fig. 1e) was the only method that effectively disrupted the cell wall of *P. glacialis*, and 10 min was sufficient to achieve complete lysis of the cells (the data for the other timepoints are not shown, but included in the OSF for this study). Neither manual grinding (Fig. 1b), microwave (Fig. 1c) or ultrathurax (Fig. 1f) was effective at disrupting the cell walls of *P. glacialis.* Sonication for 10 min was therefore chosen as the cell disruption method for lipid extraction.

### Total lipid yields

**Figure 2:**
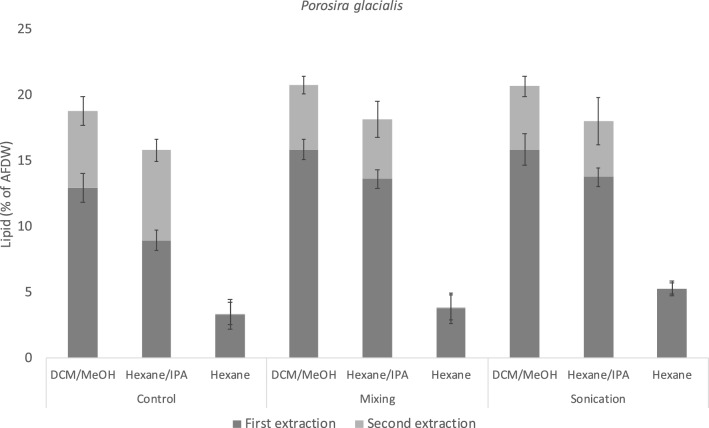
Lipid yields as percent of AFDW (Ash-free dry weight) from *Porosira glacialis* using three solvent systems; dichloromethane/methanol (2:1 v/v); DCM/MeOH), Hexane/isopropanol (2:1 v/v; Hexane/IPA) and hexane; and three cell disruption treatments; no treatment (control), shaking at 1000 RPM for 60 min (mixing) and sonication at 20 kHz for 10 min (sonication). Two consecutive extractions were performed, data shown is the arithmetic mean of each extraction, n = 5. The error bars represent the standard deviation of the mean for the first extraction (bottom bar) and for the total yield (top bar).

In *P. glacialis,* the solvent DCM/MeOH gave higher total lipid yields than both hexane/IPA and hexane independently of treatment, and the differences were statistically significant for both extractions (Fig. 2). The highest lipid yields overall was achieved when extracting with DCM/MeOH using mixing and sonication, both for the first extraction (15.9% for both treatments) and for the total yield after two extractions (20.7% for both treatments). The lipid yield in the control sample was significantly lower (p < 0.05) compared to mixing and sonication for both extractions in DCM/MeOH (13.0% and 18.8%, respectively).

Hexane/IPA was almost as effective as DCM/MeOH in extracting lipids from *P. glacialis*. The total lipid yield was approximately 18.1% of AFDW after both mixing and sonication and as for DCM/MeOH, this was significantly higher than in the sample with no treatment (Fig. 2). Extracting with hexane resulted in much lower lipid yields independently of treatment compared to both DCM/MeOH and hexane/IPA, with a maximum yield of 5.3% in the sonicated samples. Hexane was also the only solvent in which sonication resulted in a significantly higher yield compared to mixing (3.9%).

Performing a second extraction had a significant effect on the total lipid yields with both DCM/MeOH and hexane/IPA, independently of treatment. In contrast, the effect of the second extraction in hexane was negligible.

Cell disruption by sonication did not increase the total lipid yields when using the polar solvents compared with mixing, despite the clear disruptive effect of the treatment (Fig. 1). This result is contrary to those found in studies on green algae and cyanobacteria^7,30,31^ and *Nannochloropsis* sp.^9^, and shows that lipids in *P. glacialis* are more accessible for extraction compared to other commonly mass-cultivated microalgae. Our results therefore indicate that solvent choice and number of extractions are the main factors that determine lipid yield in lyophilized material of *P. glacialis*.

**Figure 3:**
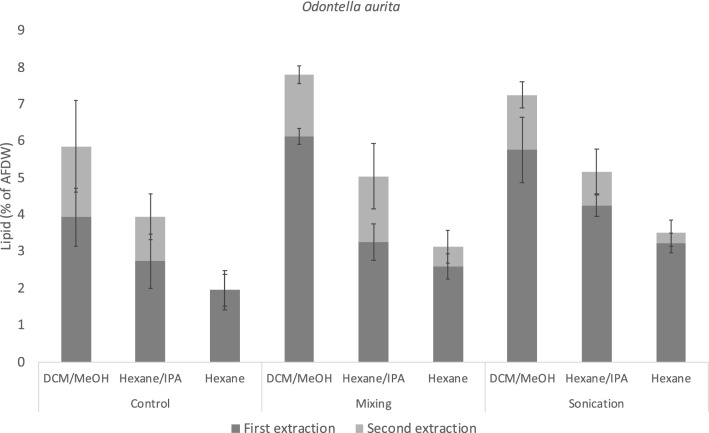
Lipid yields as percent of AFDW (Ash-free dry weight) from *Odontella aurita* using three solvent systems; dichloromethane/methanol (2:1 v/v) (DCM/MeOH), hexane/isopropanol (2:1 v/v) (Hexane/IPA) and hexane; and three cell disruption treatments; no treatment (control), mixing at 1000 RPM for 60 min (mixing) and sonication at 20 kHz for 10 min (sonication). Two consecutive extractions were performed, data shown is the arithmetic mean of each extraction, n = 5. The error bars represent the standard deviation of the mean for the first extraction (bottom bar) and for the total yield (top bar).

The highest lipid yield in *O. aurita* was achieved with DCM/MeOH in combination with mixing (7.8%), although this result was not statistically different from the yield when extracted with DCM/MeOH in combination with sonication (7.3%) (Fig. 3). Hexane/IPA was less effective, resulting in a maximum yield of 5.2% in the sonicated samples. Hexane was the least effective solvent in *O. aurita*, with a maximum lipid yield of 3.5% in the sonicated samples. Performing a second extraction had a significant effect on the total lipid yields for all three solvents with the exception of hexane in combination with control.

DCM/MeOH was a more effective solvent than both hexane/IPA and hexane when extracting lipids from both *P. glacialis* and *O. aurita*. Other studies have found that hexane/IPA can replace chlorinated solvent mixtures when extracting specific lipids from bacteria^32^ and rat brain^19^. However, a study on fish meal found that hexane/IPA gave lower lipid yields compared to chloroform-based methods, probably due the lower polarity of hexane/IPA^33^. Likewise, our results indicate that Hexane/IPA is not an ideal substitute for chlorinated solvents when extracting lipids from diatom biomass in laboratory-scale extractions. With that being said, the effect of replacing DCM/MeOH with hexane/IPA was less severe for *P. glacialis.* Extracting with hexane/IPA resulted in a 33.3% lower lipid yield in *O. aurita,* and only 12.1% in *P. glacialis* compared to the highest yield achieved with DCM/MeOH. Our results therefore indicate that hexane/IPA can be used as an alternative to chlorinated solvent mixtures in large-scale lipid extractions from *P. glacialis* with a minor loss of product recovery.

**Figure 4:**
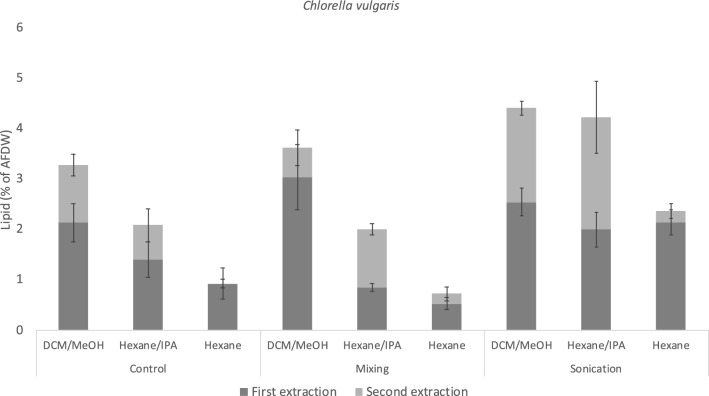
Lipid yields as percent of AFDW (Ash-free dry weight) from *Chlorella vulgaris* using three solvent systems; dichloromethane/methanol (2:1 v/v) (DCM/MeOH), Hexane/isopropanol (2:1 v/v) (Hexane/IPA) and hexane; and three cell disruption treatments; no treatment (control), mixing at 1000 RPM for 60 min (mixing and sonication at 20 kHz for 10 min (sonication). Two consecutive extractions were performed, data shown is the arithmetic mean of each extraction, n = 5. The error bars represent the standard deviation of the mean for the first extraction (bottom bar) and for the total yield (top bar).

In contrast to the two diatoms, the highest yield in *C. vulgaris* was dependent on treatment, not solvent (Fig. 4). Sonication in combination with DCM/MeOH and hexane/IPA resulted in the highest yields (4.4% and 4.2%, respectively). Mixing did not increase the total yield significantly (p > 0.05) compared to the control with any of the three solvents. Based on the first extraction alone, the lipid yield when extracting with hexane was not statistically different (p > 0.05) to the yield when using DCM/MeOH and hexane/IPA in the sonicated samples. However, the second extraction increased the yield significantly with both DCM/MeOH and hexane/IPA independently of treatment. The effect of the second extraction was much lower in hexane. Our results indicate that hexane/IPA can replace DCM:MeOH when extracting lipids from *C. vulgaris,* if used in combination with sonication. Hexane was not an effective solvent for lipid extraction in any of the three microalgae tested in this experiment, despite applying sonication to disrupt the cell walls, and should be avoided with the objective of maximizing yield. All numerical values are provided in the Open Science Framework supplied with this study.

### Lipid class composition

In *P. glacialis,* the distribution of lipid classes was highly dependent on solvent polarity (Table 1). The lipids extracted with DCM/MeOH and hexane/IPA were dominated by the polar membrane lipids MGDG, PG and PC, including some DGDG in the sonicated samples. The lipids extracted with hexane primarily contained the neutral lipids TAG, DAG and FFA. The content of FFA in *P. glacialis* was dependent on solvent, with a minimum of about 9% in the DCM/MeOH extracts and a maximum of 40% with hexane. The high content of FFA in the hexane extracts is because only the more hydrophobic lipids, not membrane lipids, are present.

**Table 1:** The relative composition of lipid classes in extracts of *Porosira glacialis*, *Odontella aurita* and *Chlorella vulgaris* extracted with dichloromethane/methanol (2:1 v/v; DCM/MeOH), hexane/isopropanol (2:1 v/v; Hexane/IPA) and hexane, using three pre-treatments; no treatment (control), shaking at 1000 RPM for 60 min, (mixing), or sonication at 20 kHz for 10 min (sonication).

Class	DCM/MeOH			Hexane/IPA			Hexane		
	Control	Mixing	Sonication	Control	Mixing	Sonication	Control	Mixing	Sonication
***P. glacialis***									
TAG	5.66 ± 0.20	5.55 ± 0.18	5.97 ± 0.37	7.92 ± 0.75	7.12 ± 0.42	8.14 ± 0.55	52.61 ± 5.56	46.12 ± 4.38	43.29 ± 3.13
DAG	10.56 ± 0.54	12.25 ± 0.62	17.38 ± 0.77	14.84 ± 0.96	15.64 ± 1.11	19.86 ± 1.57	9.13 ± 0.78	12.19 ± 2.57	20.80 ± 3.33
FFA	9.81 ± 0.70	9.31 ± 2.43	8.67 ± 1.07	12.27 ± 0.75	12.71 ± 1.75	14.02 ± 1.72	37.15 ± 6.28	40.48 ± 6.31	33.72 ± 5.82
MGDG	40.06 ± 1.09	36.30 ± 2.62	26.92 ± 1.84	36.69 ± 1.58	35.56 ± 1.08	33.28 ± 5.07	1.11 ± 0.57	1.21 ± 0.48	2.18 ± 0.28
DGDG	0	0	8.52 ± 0.46	0	0	0	0	0	0
PG	19.10 ± 0.41	21.6 ± 2.32	0	15.95 ± 1.18	14.29 ± 0.86	0	0	0	0
PC	14.81 ± 0.68	14.95 ± 2.19	32.55 ± 1.21	12.33 ± 1.95	14.68 ± 1.17	24.70 ± 2.51	0	0	0
***O. aurita***									
TAG	4.39 ± 0.28	8.27 ± 0.93	7.45 ± 0.41	3.70 ± 0.77	4.23 ± 0.46	5.75 ± 0.93	3.92 ± 0.48	3.59 ± 0.56	5.93 ± 0.61
DAG	3.05 ± 0.14	4.24 ± 0.58	3.58 ± 0.15	3.98 ± 0.62	3.53 ± 0.43	3.78 ± 0.52	3.71 ± 0.43	2.57 ± 0.39	4.39 ± 0.26
FFA	70.81 ± 3.48	71.81 ± 3.79	74.67 ± 1.67	78.26 ± 3.63	80.70 ± 1.91	76.26 ± 4.35	92.37 ± 0.91	93.84 ± 0.92	89.68 ± 0.77
MAG	2.55 ± 0.31	3.14 ± 0.44	2.79 ± 0.31	2.49 ± 0.30	2.64 ± 0.27	2.78 ± 0.40	0	0	0
MGDG	0.39 ± 0.08	0.89 ± 0.16	1.06 ± 0.27	0.54 ± 0.36	0.47 ± 0.21	1.22 ± 0.48	0	0	0
PC	18.81 ± 3.33	11.64 ± 5.04	10.45 ± 1.64	11.03 ± 2.18	8.43 ± 1.13	10.21 ± 4.96	0	0	0
***C. vulgaris***									
TAG	17.42 ± 1.02	15.88 ± 1.49	19.11 ± 0.82	4.09 ± 0.33	9.00 ± 1.38	21.07 ± 2.04	20.87 ± 3.68	39.39 ± 2.08	45.84 ± 2.44
DAG	9.69 ± 0.45	9.38 ± 0.79	10.29 ± 0.51	5.72 ± 0.56	7.81 ± 0.34	11.67 ± 0.94	5.56 ± 1.13	10.18 ± 0.55	15.80 ± 0.72
FFA	39.31 ± 2.21	38.40 ± 2.32	26.33 ± 1.18	54.08 ± 2.53	45.52 ± 2.10	25.06 ± 2.93	58.42 ± 3.66	39.76 ± 2.48	12.40 ± 1.31
MGDG	15.06 ± 1.39	13.76 ± 0.75	19.54 ± 0.78	17.53 ± 2.67	21.27 ± 0.75	22.67 ± 2.10	0	0	3.23 ± 0.42
DGDG	0	0	3.08 ± 0.12	0	0	2.50 ± 0.28	0	0	0
PC	18.52 ± 1.23	22.58 ± 2.75	21.65 ± 1.95	18.57 ± 4.48	16.40 ± 1.72	17.04 ± 1.20	15.15 ± 5.56	10.67 ± 1.24	22.73 ± 3.97

Data shown is averages of replicates ± SD, n = 5.

*TAG* triacylglycerol, *DAG* diacylglycerol, *FFA* free fatty acid, *MAG* monoacylglycerol, *MGDG* monogalactosyldiacylglycerol, *DGDG* digalactosyldiacylglycerol, *PG* phosphatidylglycerol, *PC* phosphatidylcholine.

Sonication of *P. glacialis* in combination with DCM/MeOH resulted in a reduction in the relative amount of MGDG and PG, and an increase in the relative amount of PCand DGDG compared to the control and mixing treatments. These 4 lipid classes are associated with the thylakoid membranes of the chloroplasts^34^, and one might expect sonication to result in higher yields of all 4 classes if the membranes are effectively disrupted by the treatment. The observed reduction of PG and MGDG is therefore difficult to explain, but could tentatively be caused by enzymatic hydrolysis of these lipids and subsequent enzymatic oxidation of polyunsaturated fatty acids to aldehydes^12,35^. However, there was no increase in the free fatty acids (Table 1) or change in the amount of PUFA in the sonicated samples (see “Fatty acid composition”).

The composition of the lipid classes in *O. aurita* and *C. vulgaris* was different from what was found in the *P. glacialis* samples. The most striking difference was the low levels of membrane lipids and the very high concentrations of free fatty acids in the extracts from the two commercially available microalgae. It is tempting to suggest that these values are interconnected, i.e. primarily the membrane lipids, with the exception of PC, have been hydrolyzed to free fatty acids. The rapid formation of FFA during storage has been well-documented in microalgae^36,37^, and both phospho- and glycolipids are targets of lipase activity in the marine diatom *Skeletonema costatum*^38^ and the haptophyte *T-Isochrysis lutea*^39^. Based on the reduction in specific lipid classes, our results indicate that enzymatic lipid hydrolysis is primarily targeted at the thylakoid-associated lipids (i.e. MGDG, DGDG, PG), and not PC, which is also associated with extra-chloroplastic membranes.

Studies have found that high levels of FFA reduces the oxidative stability of vegetable and marine oils by acting as a prooxidant^40,41^. The high levels of FFA may have resulted in a reduction in the observed amount of PUFA in our samples, as the rate of oxidation is positively correlated with the degree of unsaturation. These results demonstrate the importance of post-harvest preservation methods of microalgal biomass, which should be investigated further. The relative composition of the other lipid classes in *O. aurita* and *C. vulgaris* was dependent on both solvent and treatment. The relative content of TAG was in most cases highest in the sonicated samples, which is probably a result of increased lipid diffusion due to cell lysis. None of the polar lipids were detected in the samples extracted with hexane, with the exception of PC in *C. vulgaris*.

### Fatty acid composition

The relative composition of fatty acids in the lipids extracted from *P. glacialis* was similar in all samples, both when comparing treatments and solvents (Table 2). The dominating fatty acids were C20:5n-3 and C16:4n-1, each contributing approximately 30% in all samples, while docosahexaenoic acid (C22:6n-3, DHA) contributed only 2–3%. Previous studies on this species found similar levels of EPA and DHA, but lower contributions of C16:4n-1^23,26^. The sum of SFA and PUFA was similar across all treatments and solvents. In general, there were no clear effects of treatment on the fatty acid composition of *P. glacialis* when comparing the extracts obtained with the polar solvents (DCM/MeOH and hexane/IPA) and the non-polar solvent (hexane), despite large differences in the lipid class composition. This indicates a homogenous distribution of fatty acids among the lipid classes in this diatom.

**Table 2:** The relative content (%) of fatty acids from Porosira glacialis extracted in dichloromethane/methanol (2:1 v/v; DCM/MeOH), hexane/isopropanol (2:1 v/v; hexane/IPA) and hexane, using three pre-treatments; no treatment (control), shaking at 1000 RPM for 60 min, (mixing), or sonication at 20 kHz for 10 min (sonication).

*P. glacialis*	DCM/MeOH			Hexane/IPA			Hexane		
FA	Control	Mixing	Sonication	Control	Mixing	Sonication	Control	Mixing	Sonication
C14:0	4.60 ± 0.42	4.63 ± 0.38	4.94 ± 0.32	4.45 ± 0.10	4.51 ± 0.35	5.30 ± 0.33	4.22 ± 0.12	4.54 ± 0.18	4.38 ± 0.15
C16:0	7.03 ± 3.81	7.33 ± 3.43	5.86 ± 0.57	5.48 ± 0.13	6.67 ± 2.38	5.91 ± 0.31	5.43 ± 0.17	5.70 ± 0.24	6.06 ± 0.12
C16:1n-7	11.16 ± 1.02	11.45 ± 0.84	11.89 ± 0.33	11.89 ± 0.16	11.44 ± 0.45	11.94 ± 0.23	13.43 ± 0.13	13.39 ± 0.42	13.23 ± 0.07
C16:2n-4	3.21 ± 0.56	3.24 ± 0.49	3.48 ± 0.05	3.36 ± 0.06	3.20 ± 0.27	3.40 ± 0.06	2.90 ± 0.02	3.95 ± 2.25	2.86 ± 0.02
C16:3n-4	6.32 ± 1.12	6.42 ± 0.96	6.91 ± 0.11	7.09 ± 0.15	6.87 ± 0.61	7.09 ± 0.15	5.87 ± 0.01	5.78 ± 0.17	5.97 ± 0.06
C16:4n-1	31.38 ± 4.62	30.79 ± 3.32	29.53 ± 0.46	30.32 ± 0.64	30.86 ± 1.75	29.87 ± 0.71	28.28 ± 0.11	27.72 ± 0.75	27.73 ± 0.31
C18:4n-3	4.69 ± 0.73	4.71 ± 0.63	4.93 ± 0.07	4.90 ± 0.10	4.63 ± 0.32	4.94 ± 0.07	5.37 ± 0.02	5.17 ± 0.14	5.09 ± 0.07
C20:5n-3	28.68 ± 5.24	29.18 ± 4.55	30.73 ± 0.51	30.31 ± 0.64	28.47 ± 2.58	29.43 ± 0.43	31.09 ± 0.21	30.99 ± 1.06	30.90 ± 0.15
C22:6n-3	2.93 ± 1.54	2.26 ± 1.81	1.73 ± 1.85	2.20 ± 1.73	3.34 ± 0.26	2.12 ± 1.64	3.41 ± 0.07	2.76 ± 1.40	3.79 ± 0.08
SFA	11.63 ± 4.21	11.96 ± 3.73	10.80 ± 0.88	9.93 ± 0.12	11.19 ± 2.72	11.20 ± 0.61	9.79 ± 0.32	10.24 ± 0.36	10.44 ± 0.25
PUFA	77.21 ± 3.24	76.59 ± 3.09	77.30 ± 1.18	78.19 ± 0.23	77.37 ± 2.28	76.86 ± 0.77	76.78 ± 0.36	76.37 ± 0.77	76.34 ± 0.32

Data shown is averages of replicates ± SD, n = 5.

*SFA* saturated fatty acids, *PUFA* polyunsaturated fatty acids.

The dominating fatty acids in *O. aurita* were C16:0 and C16:1n-7, with a combined contribution of more than 60% in all samples (Table 3). In total, saturated and monounsaturated fatty acid made up about 80% of all fatty acids in this diatom. In contrast to *P. glacialis*, eicosapentaenoic acid (C20:5n-3, EPA) contributed only 11% of the fatty acids in all extracts of *O. aurita,* but the content of DHA was similar (2–3%). A previous study on *O. aurita* found a comparable fatty acid composition when the algae was cultivated at 24 °C, but not at lower temperatures^42^.

**Table 3:** The relative content (%) of fatty acids from Odontella aurita extracted in dichloromethane/methanol (2:1 v/v; DCM/MeOH), hexane/isopropanol (2:1 v/v; hexane/IPA) and hexane, using three pre-treatments; no treatment (control), shaking at 1000 RPM for 60 min, (mixing), or sonication at 20 kHz for 10 min (sonication).

*O. aurita*	DCM/MeOH			Hexane/IPA			Hexane		
FA	Control	Mixing	Sonication	Control	Mixing	Sonication	Control	Mixing	Sonication
C14:0	11.29 ± 0.45	12.15 ± 0.12	12.46 ± 0.29	12.09 ± 0.23	12.26 ± 0.16	12.64 ± 0.15	11.81 ± 0.32	12.12 ± 0.19	12.10 ± 0.12
C16:0	27.14 ± 0.33	26.18 ± 0.18	26.10 ± 0.20	27.49 ± 0.29	27.38 ± 0.27	26.88 ± 0.13	28.84 ± 0.25	28.25 ± 0.24	27.91 ± 0.25
C16:1n-7	36.51 ± 0.65	35.71 ± 0.30	35.68 ± 0.29	37.23 ± 0.14	37.11 ± 0.37	36.06 ± 0.40	37.86 ± 0.26	38.60 ± 0.10	37.31 ± 0.22
C16:2n-4	3.03 ± 0.09	3.10 ± 0.02	3.04 ± 0.03	2.96 ± 0.02	2.96 ± 0.02	2.96 ± 0.04	2.93 ± 0.02	2.83 ± 0.01	2.81 ± 0.04
C16:3n-4	2.28 ± 0.07	2.35 ± 0.01	2.30 ± 0.02	2.02 ± 0.04	2.07 ± 0.03	2.10 ± 0.04	1.80 ± 0.02	1.62 ± 0.01	1.67 ± 0.05
C18:0	1.11 ± 0.03	1.20 ± 0.02	1.25 ± 0.05	1.21 ± 0.12	1.41 ± 0.07	1.53 ± 0.13	1.22 ± 0.07	1.40 ± 0.04	1.62 ± 0.04
C18:1n-9	2.98 ± 0.06	3.11 ± 0.03	3.02 ± 0.02	2.70 ± 0.05	2.77 ± 0.05	2.82 ± 0.06	2.47 ± 0.02	2.29 ± 0.03	2.31 ± 0.08
C18:1n-7	1.60 ± 0.02	1.94 ± 0.02	1.81 ± 0.02	1.63 ± 0.09	1.57 ± 0.10	1.58 ± 0.12	1.13 ± 0.02	1.17 ± 0.01	1.15 ± 0.01
C18:2n-6	1.21 ± 0.05	1.27 ± 0.05	1.25 ± 0.01	1.13 ± 0.01	1.20 ± 0.03	1.20 ± 0.03	1.04 ± 0.04	1.12 ± 0.05	1.13 ± 0.03
C20:5n-3	10.96 ± 0.17	12.35 ± 0.15	11.56 ± 0.12	10.03 ± 0.15	10.17 ± 0.15	11.14 ± 0.09	9.77 ± 0.07	10.11 ± 0.11	10.67 ± 0.20
C22:6n-3	1.89 ± 1.25	0.65 ± 0.72	1.52 ± 0.70	1.52 ± 0.03	1.08 ± 0.64	1.09 ± 0.80	1.14 ± 0.52	0.47 ± 0.45	1.32 ± 0.62
SFA	39.54 ± 0.29	39.53 ± 0.31	39.82 ± 0.34	40.79 ± 0.17	41.05 ± 0.29	41.05 ± 0.27	41.86 ± 0.31	41.77 ± 0.34	41.63 ± 0.39
MUFA	41.09 ± 0.73	40.76 ± 0.31	40.51 ± 0.31	41.56 ± 0.14	41.46 ± 0.31	40.46 ± 0.49	41.46 ± 0.29	42.06 ± 0.11	40.77 ± 0.28
PUFA	19.37 ± 0.99	19.72 ± 0.61	19.67 ± 0.64	17.65 ± 0.19	17.49 ± 0.54	18.50 ± 0.64	16.68 ± 0.54	16.17 ± 0.38	17.61 ± 0.58

Data shown is averages of replicates ± SD, n = 5.

*SFA* saturated fatty acids, *MUFA* monounsaturated fatty acids, *PUFA* polyunsaturated fatty acids.

The relative composition of the individual fatty acids and the total amount of SFA, MUFA and PUFA showed little variation across both solvent and treatment (Table 3). The dominating fatty acids in *C. vulgaris* were C16:0, C18:2n-6 and C18:3n-3, totaling approximately 80% of the fatty acids in all samples (Table 4). In contrast to *P. glacialis* and *O. aurita*, *C. vulgaris* did not contain any fatty acids of more than 18 carbons, which agrees with other studies on *C. vulgaris*^43,44^. The changes in lipid class composition of *O. aurita* and *C. vulgaris* as a result of solvent and treatment were accompanied by changes in the fatty acid composition; the relative content of PUFA was higher in DCM/MeOH compared to hexane. This indicates that the relative content of PUFAs is higher in the membrane-associated lipids in *O. aurita* and *C. vulgaris*.

**Table 4:** The relative content (%) of fatty acids from Chlorella vulgaris extracted in dichloromethane/methanol (2:1 v/v; DCM/MeOH), hexane/isopropanol (2:1 v/v; hexane/IPA) and hexane, using three pre-treatments; no treatment (control), shaking at 1000 RPM for 60 min, (mixing), or sonication at 20 kHz for 10 min (sonication).

*C. vulgaris*	DCM/MeOH			Hexane/IPA			Hexane		
FA	Control	Mixing	Sonication	Control	Mixing	Sonication	Control	Mixing	Sonication
C16:0	24.79 ± 0.55	24.91 ± 0.17	23.25 ± 0.08	30.03 ± 5.95	25.42 ± 0.03	27.41 ± 2.75	24.63 ± 0.6	26.69 ± 0.2	29.29 ± 5.05
C16:3n-4	10.40 ± 0.21	10.61 ± 0.07	10.80 ± 0.08	9.93 ± 1.32	10.62 ± 0.25	9.79 ± 0.65	12.18 ± 0.21	10.80 ± 0.24	8.95 ± 0.99
C18:0	1.49 ± 0.09	2.01 ± 0.10	1.49 ± 0.12	2.47 ± 0.56	2.86 ± 0.28	1.95 ± 0.15	6.11 ± 0.13	8.30 ± 0.11	2.80 ± 0.61
C18:1n-9	4.01 ± 0.12	3.96 ± 0.06	3.96 ± 0.04	3.47 ± 0.07	3.74 ± 0.08	4.02 ± 0.05	3.63 ± 0.01	4.46 ± 0.08	4.85 ± 0.15
C18:1n-7	1.10 ± 0.01	1.09 ± 0.01	1.04 ± 0.01	1.00 ± 0.03	1.10 ± 0.02	1.05 ± 0.01	0	0.92 ± 0.01	1.07 ± 0.04
C18:2n-6	37.34 ± 0.19	36.51 ± 0.02	37.53 ± 0.07	33.89 ± 3.02	35.29 ± 0.2	35.63 ± 1.27	32.93 ± 0.22	30.00 ± 0.04	33.88 ± 2.7
C18:3n-3	20.87 ± 0.25	20.90 ± 0.09	21.93 ± 0.03	19.21 ± 2.15	20.97 ± 0.25	20.15 ± 1.02	20.52 ± 0.31	18.83 ± 0.04	19.16 ± 1.65
SFA	26.28 ± 0.60	26.92 ± 0.27	24.74 ± 0.16	32.50 ± 6.50	28.28 ± 0.57	29.36 ± 2.88	30.74 ± 0.73	34.99 ± 0.32	32.10 ± 5.52
MUFA	5.11 ± 0.13	5.06 ± 0.05	5.00 ± 0.05	4.47 ± 0.09	4.84 ± 0.10	5.07 ± 0.06	3.63 ± 0.01	5.38 ± 0.10	5.92 ± 0.19
PUFA	68.61 ± 0.61	68.02 ± 0.25	70.27 ± 0.14	63.03 ± 6.47	66.88 ± 0.65	65.57 ± 2.92	65.63 ± 0.74	59.63 ± 0.23	61.99 ± 5.34

Data shown is averages of replicates ± SD, n = 5.

*SFA* saturated fatty acids, *MUFA* monounsaturated fatty acids, *PUFA* polyunsaturated fatty acids.

## Conclusions

DCM/MeOH is a better solvent than hexane and hexane/IPA for extracting lipids from *P. glacialis.* However, hexane/IPA also works well and is a better alternative in large-scale extractions. Sonication did not increase the lipid yield or influence the fatty acid composition in *P. glacialis* and *O. aurita* compared to mixing. Cell wall disruption is therefore not a prerequisite to obtain high product yields in *P. glacialis* and probably diatoms in general, in contrast to other mass-cultivated microalgae. In conclusion, choice of solvent and number of extractions were the main factors that determined lipid yield and composition in *P. glacialis.*

## Supplementary information

Supplementary Table S1

## Data Availability

The raw data obtained in this study along with the R scripts used for analysis and graphing are available from the Open Science Framework (OSF) under the name “Choice of solvent and number of extractions are the main factors that determine lipid yield in a marine centric diatom” at https://osf.io/sxrvz/?view_only=aa4342d55dd348768ed77ac06aee7c97.
