# Supplementary material for: Lipid yield from the diatom Porosira glacialis is determined by solvent choice and number of extractions, independent of cell disruption
Source: Sci Rep. 2020 Dec 17;10:22229. doi: 10.1038/s41598-020-79269-z (PMC7747635; doi:10.1038/s41598-020-79269-z)
Supplement: Supplementary file 1 — Supplementary Table S1 [file 41598_2020_79269_MOESM1_ESM.docx]

Supplementary information

# Title: Lipid yield from the diatom *Porosira glacialis* is determined by solvent choice and number of extractions, independent of cell disruption

Jon Brage Svenning ^1*^, Lars Dalheim^1^, Terje Vasskog^2^, Lucie Matricon^3^, Birthe Vang^3^, Ragnar Ludvig Olsen^1^

^1^ Norwegian College of Fishery Science, UiT The Arctic University of Norway, 9037 Tromsø; jon.b.svenning@uit.no; lars.dalheim@uit.no; ragnar.olsen@uit.no

^2^ Department of Pharmacy, Faculty of Health Sciences, UiT The Arctic University of Norway, 9037 Tromsø; Terje.Vasskog@uit.no

^3^ Nofima AS, Muninbakken 9-13, Breivika, 9019 Tromsø; Lucie.matricon@gmail.com; Birthe.vang@nofima.no

*Supplementary table S1: Solvent gradient program used for normal-phase liquid chromatography of lipid classes. Mobile phase A = isooctane/ethyl acetate (99.8:0.2), Mobile phase B = acetone/ethyl acetate (2:1) 0.15 % acetic acid. Mobile phase C = isopropanol/H_2_O (85:15)*

| Time (min) | Solvent A | Solvent B | Solvent C | Flow (ml min^-1)^ | Curve |
| --- | --- | --- | --- | --- | --- |
| 0.0 | 100 | 0 | 0 | 1.5 | 1 |
| 1.5 | 100 | 0 | 0 | 1.5 | 6 |
| 1.6 | 97 | 3 | 0 | 1.5 | 6 |
| 6.0 | 94 | 6 | 0 | 1.5 | 6 |
| 8.0 | 50 | 50 | 0 | 1.5 | 6 |
| 8.1 | 46 | 39 | 15 | 1.5 | 6 |
| 14.0 | 43 | 30 | 27 | 1.5 | 6 |
| 14.1 | 43 | 30 | 27 | 1.5 | 6 |
| 18.0 | 40 | 0 | 60 | 1.5 | 6 |
| 23.0 | 40 | 0 | 60 | 1.5 | 6 |
| 24.0 | 0 | 100 | 0 | 1.5 | 6 |
| 25.0 | 0 | 100 | 0 | 2.0 | 6 |
| 27.0 | 0 | 100 | 0 | 2.0 | 6 |
| 27.1 | 100 | 0 | 0 | 2.0 | 6 |
| 36.0 | 100 | 0 | 0 | 2.0 | 6 |
| 36.1 | 100 | 0 | 0 | 1.5 | 6 |
